# Infantile-onset inflammatory bowel disease has variable long-term outcomes

**DOI:** 10.3389/fped.2023.1097779

**Published:** 2023-03-01

**Authors:** Alex Krauthammer, Ilana Weintraub, Ron Shaoul, Raffi Lev-Tzion, Efrat Broide, Michael Wilschanski, Aaron Lerner, Baruch Yerushalmi, Dror S. Shouval, Hussein Shamaly, Yael Haberman-Ziv, Batia Weiss

**Affiliations:** ^1^ Pediatric Gastroenterology Unit, Edmond and Lily Safra Children's Hospital, Sheba Medical Center, Tel hashomer, Israel; ^2^ Sackler Faculty of Medicine, Tel-Aviv University, Tel Aviv, Israel; ^3^ Pediatric Gastroenterology Unit, Faculty of Medicine, Ruth Rappaport Children’s Hospital, Rambam Health Care Campus, Haifa, Israel; ^4^ Paediatric Gastroenterology, Shaare Zedek Medical Centre, Jerusalem, Israel; ^5^ Pediatric Gastroenterology Unit, Shamir Medical Center, Sackler Faculty of Medicine Tel Aviv University, Tel Aviv, Israel; ^6^ Pediatric Gastroenterology Unit, Hadassah Hebrew University Hospital, Jerusalem, Israel; ^7^ Pediatric Gastroenterology and Nutrition Unit, Carmel Medical Center, B, Rappaport School of Medicine, Technion-Israel Institute of Technology, Haifa, Israel; ^8^ Pediatric Gastroenterology Unit, Soroka University Medical Center, Faculty of Health Sciences, Ben-Gurion University of the Negev, Beer-Sheva, Israel; ^9^ Institute of Gastroenterology, Nutrition and Liver Diseases, Schneider Children's Medical Center, Sackler Faculty of Medicine Tel-Aviv University, Tel Aviv, Israel; ^10^ Department of Pediatrics, Saint Vincent de Paul-French Hospital, Nazareth, Israel

**Keywords:** inflammatory bowel disease, infantile onset, remission, surgery, long-term outcome

## Abstract

**Objective and aim:**

Infantile-onset inflammatory bowel disease (IO-IBD), defined as IBD diagnosed at age 2 years or younger, tends to be more severe and refractory to conventional treatment than IBD diagnosed at a later age. However, data about IO-IBD and its long-term follow up are limited. We thus aimed to evaluate the presentation and long-term outcomes of patients with IO-IBD in a retrospective multicenter study.

**Methods:**

Medical records of patients diagnosed with IO-IBD in eight medical centers during 2000–2017 with at least 1-year follow up were reviewed. Demographics and disease characteristics at diagnosis including age of onset, disease phenotype and location, surgeries, medical therapy, and comorbid conditions were recorded.

**Results:**

Twenty-three patients with IO-IBD (16 males, 70%) were identified and followed for a median (range) of 51.2 (26.0–110.3) months. The mean ages at presentation and at the last follow up were 14 ± 9.8 and 101 ± 77 months, respectively. Six (26%) patients needed ileostomy already at the time of diagnosis and 20 (87%) were treated with corticosteroids. During long-term follow up, remission was achieved in 16 (73%) patients; of whom, 3 (14%) were without medications and 7 (32%) were in remission with the use of 5-aminosalicylic acid only. One patient needed hemicolectomy and one developed a severe EBV related infection.

**Conclusion:**

The majority of patients with IO-IBD achieved long-term remission, despite a severe disease presentation at diagnosis. Surgery rate however is high, mainly during the first months from diagnosis.

## Introduction

Very early onset inflammatory bowel disease (VEO-IBD), defined as disease onset at age 6 years or younger, represents 6%–15% of pediatric IBD incidences (1, 2). Two subclassifications of VEO-IBD are neonatal IBD, defined as disease appearance in the first 28 days of life, and infantile-onset IBD (IO-IBD), defined when symptoms’ onset is at age 2 years or younger (3–6). Studies of VEO-IBD, have reported inconclusive disease course and natural history compared to IBD diagnosed at a later age (5–7). IO-IBD differs from IBD diagnosed at older ages by a predominant colonic involvement, an association with monogenic diseases (10%–20%), a high rate of positive family history of IBD, and poor response to therapy (2, 7–19). Prior studies identified that IO-IBD patients require more aggressive medical treatment and have higher rate of surgical intervention compared to later onset disease (2, 12, 13, 20). Data on long-term outcomes of IO-IBD are scarce (17, 21). Small-scale studies reported variable outcomes, ranging from high rates of surgery to complete remission during follow-up (21, 22). We aimed to report the long-term outcome of a cohort of patients with IO-IBD in a retrospective, multi-center study.

## Materials and methods

The databases of children with IBD, followed at eight medical centers in Israel from January 2000 to December 2017 were reviewed. Infants with formal diagnosis of IBD at age 2 years or younger, with a follow-up period of at least 12 months, were included. IBD was diagnosed according to standard criteria (23, 24). Demographic, clinical, laboratory, endoscopic, and histological details at diagnosis and during follow up were retrieved from the children's medical records. Those with evidence of infectious or allergic colitis, and known immune deficiencies or systemic diseases presenting prior to the gastrointestinal symptoms, were excluded.

The primary outcome was clinical remission at the last visit. Secondary outcomes included the need for surgery and steroid dependency. Disease activity and clinical remission were evaluated by using the weighted Pediatric Crohn's Disease Activity Index for patients with a CD-like phenotype and the Pediatric Ulcerative Colitis Activity Index for patients with UC and IBD unclassified (IBD-U) (25, 26). Growth failure was defined as a difference of height *Z*-score >1 between the pre-illness and the last available height *Z*-score (27). Anemia was defined as a hemoglobin level <11 gr/dl, thrombocytosis as platelets >450,000, hypoalbuminemia as serum albumin <3.5 gr/dl, and C-reactive protein (CRP) elevation as >5 mg/L.

The study was approved by the institutional review board of each institution.

### Statistical analysis

The data are presented as percentages for descriptive variables; and as medians and interquartile (lower and upper) ranges (IQRs), or as means and standard deviations (SDs) for continuous variables, as appropriate. We did not perform statistical analysis between subgroups (by disease location), because of the small sample size. The data were analyzed by a biostatistician using IBM SPSS statistics version 24 (Armonk, NY: IBM Corp).

## Results

During the study period, 23 patients with IO-IBD, of whom 16 were males (70%), met the inclusion criteria. Two patients had neonatal onset IBD both diagnosed at age 1 month. The mean age at diagnosis was 14.0 ± 9.8 months; 10 (44%) patients were classified as having UC and 13 (56%) as CD (Table 1). The median duration of follow-up was 51.2 (interquartile range [IQR] 26–110) months (Table 1). The most common presenting symptoms were diarrhea (23, 100%) and hematochezia (20, 87%). Extraintestinal manifestations were present in 10 (44%). Perianal involvement was present in 7 (30%) patients, of them 2 (9%) had perianal fistulas and 5 (22%) had deep fissures. The most prevalent laboratory abnormalities at presentation were iron deficiency anemia in 16/20 (80%), thrombocytosis in 13/19 (60%), hypoalbuminemia in 10/19 (53%), and elevated CRP in 14/17 (83%) (Table 2). Whole-exome sequencing (WES), performed in 10 patients, revealed two monogenic diseases: one with an IL-10 receptor mutation and one with a CARMIL2 mutation.

**Table 1:** Demographic and clinical characteristics of children with infantile-onset inflammatory bowel disease.

Parameter	*N* = 23
**General characteristics**	
Male gender, *n* (%)	16 (70)
Gestational age (weeks), mean ± SD	39.5 ± 1.1
Birth weight (kg), median (IQR)	3.5 (2.9 to 3.7)
Family history of IBD, *n* (%)	11/20 (55)
Breastfeeding, *n* (%)	18/20 (90)
Age at diagnosis (months), mean ± SD	14.3 ± 10
Current age (months), mean ± SD	101 ± 77
Follow up time (months), median (IQR)	51.2 (26 to 110.3)
Length *Z* score—diagnosis, median (IQR)	−0.6 (−1.36 to 0.4)
Weight *Z* score—diagnosis, median (IQR)	−1.3 (−2.5 to −0.6)
**Presenting symptoms *n* (%)**	
Diarrhea	23 (100)
Hematochezia	20 (87)
Failure to thrive	11 (48)
Perianal involvement	7 (30)
Extraintestinal manifestation	10 (44)
Fever	6 (26)
Arthritis	6 (26)
**Disease phenotype *n* (%)**	
Crohn's disease	13 (57)
Ulcerative colitis	10 (43)
**Disease location *n* (%)**	
Pancolitis	14 (61)
Left-sided colitis	1 (4)
Crohn's colitis	8 (35)
Terminal Ileum	1 (4)
Upper GI tract	11/18 (61)

Kg, kilogram; IQR, interquartile range; SD, standard deviation; IBD, inflammatory bowel disease; GI, gastrointestinal.

**Table 2:** Laboratory data at presentation and initial treatment in children with infantile-onset inflammatory bowel disease.

Parameter	Value
**Laboratory**	
White blood cells (x 10^3^), mean ± SD	15.3 ± 6.4
Hemoglobin (gr/dl), mean ± SD	9.6 ± 1.4
MCV (fl), mean ± SD	70.3 ± 8.3
Platelet count (x 10^3^), mean ± SD	583 ± 165
Albumin (gr/dl), mean ± SD	3.3 ± 0.8
CRP (mg/L), median (IQR)	19 (4–83)
ESR (millimeter/hour), median (IQR)	29 (20–55)
**Serology**	
ASCA positive, *n* (%)	1/12 (8)
ANCA positive, *n* (%)	2/11 (18)
**Genetic evaluation** **^a^**	
IL-10 Receptor mutation, *n* (%)	1/10 (10)
CARMIL2 mutation, *n* (%)	1/10 (10)
**Medications****^a^** ***n* (%)**	
5-ASA	17/23 (74)
Aza/6-MP	16/23 (70)
Corticosteroids	20/23 (87)
Anti TNF-α	7/23 (30)
TPN^b^	8/16 (50)
**Surgery at first hospitalization, *n* (%)**	**6/23** **(26)**
Ileostomy	1/23 (4.3)
Ileostomy + subtotal colectomy	1/23 (4.3)
Ileostomy + total colectomy	4/23 (17.4)

MCV, mean corpuscular volume; CRP, C-reactive protein; ESR, erythrocyte sedimentation rate; ASCA, anti-saccharomyces cerevisiae antibody; ANCA, antineutrophil cytoplasmic antibody; IL, interleukin; TNF, tumor necrosis factor; Aza, azathioprine; 6-MP, 6-mercaptopurine; 5-ASA, 5-aminosalicylic acid; TPN, total parenteral nutrition.

^a^ 21/23 (91%) of the patients were treated with more than one medication.

^b^ Data were available for only 16 patients.

### Endoscopy and histology

All patients had colonic inflammation at diagnosis, of whom, 14 (61%) had pancolitis. Terminal ileum (TI) involvement was observed in 1 (4%) patient who was classified as CD. Esophagogastroduodenoscopy (EGD) was completed in 18 (78%) patients, of them, 11 (61%) had signs of macroscopic inflammation and histologically, chronic inflammation was absent in 8/18 (44%) patients. Classification of the 13 patients with CD included 11 with inflammatory disease (B1), one with stricturing disease (B2) and 2 with fistulizing disease (B3). Granulomas were found in intestinal biopsies of 4/13 (31%) of the CD patients.

### Medical, nutritional, and surgical therapy

The initial medical therapy after diagnosis was a combination of corticosteroids with 5-aminosalicylic acid (5-ASA) or an immunomodulatory agent [6-mercaptopurine (6-MP) or azathioprine (AZA)] in 20/23 (87%) patients. Monotherapy with infliximab, 5-ASA, or AZA was initiated in one patient each (Table 3). Following induction, an additional six patients needed escalation to biological therapy and 10/20 (50%) were steroid dependent. Information on nutritional therapy at presentation was available for 16 patients, 8 of whom were treated with total parenteral nutrition (TPN). A response to biological treatment was observed in 4/7 (57%) patients. However, in three of them treatment was discontinued due to allergic reactions (Table 3). Diverting ileostomy was performed in 6 (26%) patients, three with a CD phenotype (one with IL-10 receptor mutation) due to intractable inflammation and steroid dependency, and three with a UC phenotype (one with CARMIL 2 mutation, due to perforation during colonoscopy).

**Table 3:** Treatment during follow-up and clinical state at last follow-up of patients with IO-IBD.

Pt	Initial treatment	Follow up treatment	Treatment at last visit	Age at last visit (months)	Follow-up (months)	Surgery	Clinical remission
**Ulcerative colitis**							
1	EEN, CS, 5-ASA 6-MP/AZA,	CS^a^, 5-ASA, 6-MP/AZA	5-ASA	15	12	No	Yes
2	5-ASA, 6-MP/AZA	NA	No treatment	16	12	Ileostomy Total colectomy	Yes
3	CS, 5-ASA, 6-MP/AZA	CS^a^, 5-ASA 6-MP/AZA	5-ASA	86	78	No	Yes
4	TPN, EEN, CS, 5-ASA, 6-MP/AZA	CS^a^, 5-ASA, 6-MP/AZA	TPN, CS^a^	138.6	123.6	Ileostomy Total colectomy	No
5	CS, 5-ASA, 6-MP/AZA	CS^a^, Anti TNF-α, 5-ASA, 6-MP/AZA	6-MP/AZA, Anti TNF-α	102	88.1	Ileostomy Total colectomy	No
6^b^	TPN, CS, 5-ASA, 6-MP/AZA	MTX	6-MP/AZA, Anti TNF-α	359.2	358.3	Right hemicolectomy	Yes
7	TPN, CS, 5-ASA	CS^a^	TPN, CS^a^	20	15	No	No
8	CS, 5-ASA, 6-MP/AZA	6-MP/AZA	5-ASA	71.4	50.6	No	Yes
9	EEN, CS, 6-MP/AZA, 5-ASA	CS^a^, 6-MP/AZA, 5-ASA	5-ASA	70.1	56.2	No	Yes
10	CS, 6-MP/AZA, 5-ASA	Anti TNF-α	Anti TNF-α	69.1	51.2	No	Yes
**Crohn's disease**							
11	TPN, EEN, CS, 6-MP/AZA, 5-ASA	CS^a^, Anti TNF-α^c^, 6-MP/AZA	5-ASA	188.1	178.1	No	Yes
12	TPN, EEN, CS, 6-MP/AZA	CS^a^, Anti TNF-α^c^, 6-MP/AZA	No treatment	98.2	88.8	No	Yes
13	TPN, EEN, CS, 6-MP/AZA	CS^a^, Anti TNF-α^c^, 6-MP/AZA, CsA	CS^a^, 6-MP/AZA, CsA	15	12	Ileostomy	No
14	TPN, CS, 6-MP/AZA	CS^a^, Anti TNF-α, 6-MP/AZA	No treatment	55	36	Ileostomy Total colectomy	No
15	CS, 6-MP/AZA, 5-ASA	6-MP/AZA	6-MP/AZA, 5-ASA	29	12	No	Yes
16	CS, 5-ASA	NA	No treatment	144	120	No	Yes
17^b^	TPN, Anti TNF-α	Anti TNF-α	Anti TNF-α	NA	NA	Ileostomy Subtotal colectomy	No
18	CS, 5-ASA	5-ASA	5-ASA	168	144	No	Yes
19	CS, 5-ASA	MTX	MTX	38	26	No	Yes
20	CS, 5-ASA, 6-MP/AZA	6-MP/AZA	6-MP/AZA	128.3	110.3	No	Yes
21	CS, EEN, 6-MP/AZA, 5-ASA	6-MP/AZA, 5-ASA	6-MP/AZA, 5-ASA	62	45	No	Yes
22	CS	NA	NA	NA	NA	NA	NA
23	5-ASA	5-ASA	5-ASA	68	50	No	Yes

NA, not available; Tx., treatment; Dx., diagnosis; mo., months; Aza, azathioprine; CsA, cyclosporine A; 6-MP, 6-mercaptopurine; MTX, methotrexate; EEN, exclusive enteral nutrition.

^a^ Steroid dependent.

^b^ Patients with neonatal onset IBD.

^c^ Discontinued due to an allergic reaction.

### Long-term outcomes

Information regarding the clinical state and treatment at the end of the follow up was available for 22 patients. The median follow-up time was 51.2 (26–110.3) months and the mean patients’ age at the last follow-up was 101 ± 77 months. Height *Z*-score and weight *Z*-score, both at diagnosis and at the end of follow up, were available in 8 and 12 patients, respectively. Thus, at diagnosis, mean height and weight *Z*-score of those patients were (−0.82 ± 1.69) and (−1.77 ± 1.92), as compared to (−0.89 ± 0.98) and (−0.89 ± 1.17) at the end of follow up, (*p* = 0.46 and *p* = 0.1), respectively. Growth failure was observed in 1/8 (12.5%) patients with available height measurements at diagnosis and end of follow up. Both height *Z*-score and weight *Z*-score at diagnosis and end of follow-up were available in 7 patients. Three patients (2 with CD, 1 with UC), had both stunting (*Z*-core ≤ 2) and wasting (*Z*-score ≤ 2) at the end of follow-up. The mean final height *Z*-score of all patients with available data was −0.8 ± 1.1. In general, the weight *Z*-score showed a tendency for improvement, when final weight *Z*-score was compared to weight *Z*-score at diagnosis: −0.7 ± 1.2 vs. −1.5 ± 1.7, *p* < 0.06.

Long-term remission, [median 51.2 (26–110.3) months], was achieved in 16/22 (73%) patients, of these, one underwent colectomy and ileostomy at diagnosis and one underwent ileostomy and hemicolectomy due to perforation during bowel dilation later in the course of disease. At last follow-up, three patients (aged 1.3, 8.2, and 12.0 years, respectively, Table 3), were in clinical remission without medical treatment, two of them (#12 and #16) with no surgeries and one (#2), after ileostomy and total colectomy very early in the course of the disease. Another seven patients were in remission with 5-ASA treatment only (3 CD, 4 UC). Monotherapy with infliximab, methotrexate, or AZA/6MP was administered to one patient each, combination therapy with infliximab and AZA/6MP to one patient, and 5-ASA with AZA/6MP to one patient. Three patients (14%) remained steroid dependent. Overall, in 11/22 (50%) patients, the treatment was de-escalated or stopped during follow-up; and in 4/22 (18%), the treatment was escalated to biological agents or immunomodulators.

Five out of six patients, who underwent surgery at the time of diagnosis, were with active disease despite the surgical procedure [of them one with IL-10 receptor mutation (patient #13), one with CARMIL 2 mutation (patient #4) and one patient in whom later in the course of the disease ileitis was demonstrated on MRE (patient #5)]. These five patients were treated with TPN, steroids, cyclosporine or anti-tumor necrosis factor (TNF)-α medications. At the end of follow up, the patient with CARMIL 2 mutation was treated with Budesonide gel for upper GI inflammation and was waiting for initiation of Sirolimus. The patient with IL-10 receptor mutation had an episode of severe sepsis after a single dose of Etanercept given for his arthritis, and at the end of follow up was without regular treatment except antibiotics and topical treatment for skin manifestations. His parents refused bone marrow transplant. Four patients (Table 3) were followed for 10 years or longer; of them, three were in remission and one was steroid dependent after surgery at diagnosis.

Severe long-term complications included bowel perforation during endoscopic follow up in one patient, and an Epstein-Barr virus infection in one patient (#10), in which lymphoma was suspected and eventually ruled out after the patient was lost to follow-up in our center.

## Discussion

The main findings of the current study are the variability in the long-term outcomes of children with IO-IBD. We report long-term remission in 73% of the patients, despite a severe inflammatory course in infancy; however, steroid dependency was common and 26% required surgical intervention.

All patients in our cohort had colonic disease, classified as UC or CD, and none were classified as having IBD-U. Other studies reported various sub-classifications of disease in IO-IBD. A retrospective study of 1,370 children with early-onset IBD in the U.S. reported similar proportions of CD, UC, and indeterminate colitis (about 33% at each subgroup) in children under age 2 years (18). In contrast, a study of 62 children who presented with IBD before age 2 years reported IBD-U in 71% (22). Notably, one-third of that cohort had monogenic diseases. In a case series of six Asian patients, 3 had CD, 2 UC, and 1 IBD-U (21). Other studies reported IBD-U in 10%–28% of children with VEO-IBD (<6 years) (16, 28), though data were not presented separately for IO-IBD. A recent population-based study from Israel classified children with VEO and IO-IBD as CD or UC only, with no IBD-U classification (29). This is due to the lack of inclusion of IBD-U code by the Israeli Health Maintenance Organizations. The differences in the distribution of UC, CD, and IBD-U between studies may also be due to differences between the populations, including the proportions of patients with monogenic disease, and differences in the rate of WES testing. In addition, we might have overlooked some patients who might have fallen under classification of IBD-U, because only 78% of our patients had EGD performed and none had small bowel imaging.

Lack of chronic inflammation on biopsy, observed in 44% of our patients on EGD, might be explained by the early performance of endoscopic evaluation due to severe disease at presentation.

We found causative mutations in 2/10 (20%) patients in whom WES was performed. As genetic testing was not available and affordable during the first years of the study period, not all the patients were evaluated. Similar studies reported various rates of mutation detection in IO-IBD. In a study from China, 16/54 (30%) patients underwent genetic evaluation; 9/54 (17%) had monogenic diseases (12). Among 62 children with IO-IBD in the UK, causative mutations were detected in 31% (22). However, a number of studies of VEO-IBD that included patients with IO-IBD did not report any genetic analyses (13, 16, 18, 22). A family history of IBD was present in 55% of our patients. This is similar to reports of 18%–44% in a number of western studies (18, 22), but contrasts to the lack of family history in studies from Asia (12, 21).

The clinical presentation of our group is in line with previous studies; bloody diarrhea was the most common symptom, and anemia hypoalbuminemia and elevated CRP were the most common laboratory findings. Fecal calprotectin was not consistently available at diagnosis as in other studies of the same age group (22).

### Treatment

The most common induction treatment at presentation was corticosteroids, within the range of 80%–92% reported for other series of IO-IBD including recent study from 4 Israeli Health Maintenance Organizations (5, 24, 29). The treatment regimens included 5-ASA, AZA, 6-MP, infliximab, and nutritional support by TPN. Infliximab treatment failure was observed in all, but one of our patients during induction. This high failure rate corroborates other reports of VEO-IBD. One study reported a high rate of treatment discontinuation before week 14 (30), due to adverse effects and treatment failure. Another study reported a higher rate of treatment failure during the first year than in older children (31). Finally, recent data showed that up to 35% newly diagnosed IO-IBD patients at each year fail to respond to biologics (29). Data have not been published specifically on the response to anti-TNFα medications in IO-IBD. The lower response rate to anti-TNFα medications in younger children may reflect a higher predisposition to infections, different pharmacokinetics, or genetic background, leading to non-TNFα mediated inflammation (30, 32, 33). In addition, we did not use therapeutic drug monitoring during the study years, and a more intense treatment regimen may be needed to control inflammation in young patients.

Of our patients with available information regarding nutritional therapy, half needed TPN at presentation. This is similar to the proportion of 59% (10/17), in the Japanese cohort of VEO-IBD (15) but lower than 90% (9/10), reported among patients who were diagnosed with IBD during their first year of life (5). Notably, patients with monogenic disease were also described to have higher TPN requirements (34). In studies of older IBD patients at older ages (35), the TPN requirement for TPN was much lower. Taken together, TPN use at presentation appears to be inversely proportional to patient age at diagnosis. The young age, together with severe diarrhea and failure to thrive, and the low response rate to anti-TNFα medications, are possible causes of the high rates of TPN initiation in IO-IBD.

Surgery at an early stage after diagnosis was performed in 26% of our patients. This is within the range of 19%–50% reported by studies from Asia, England, France and Israel (5, 21, 22, 29). The need for early surgery reflects the severity of disease at presentation in this age group and was shown to have 1.4 times higher risk compared to toddler-onset group (29). Another possible explanation to high surgery rates at this age group is their biological treatment refractory “behavior” most probably due to suboptimal dosing of biological agents and unclear pharmacokinetics (30, 32, 33).

### Outcome

The long-term outcomes of the patients in our cohort were more favorable than previously described, despite the severe disease at presentation. Clinical remission was achieved in 73% of the patients after a median of 51 months. Notably, at the end of the follow up, about one-third of the patients were treated only with 5-ASA and 3 (14%) remained without any treatment, though one of them needed surgery early in the course of the disease. In other studies only 26% of children in the UK with IO-IBD did not require treatment escalation beyond first-line immunomodulator therapy (22), and 2 (20%) of 10 children diagnosed during their first year of life with IBD in France, stopped medications within 1 year and had long-term remission (5). The early surgery rate, 26% at the first hospitalization, was similar to that described in the literature for IO-IBD (7), and higher than in later-onset childhood IBD (36). Interestingly, after the initial surgeries, none of our patients required repeat surgery, except one patient who had a bowel perforation during colonoscopy. Similar to other studies of IO-IBD patients, one of our patients (#5) who was primarily diagnosed with UC and underwent total colectomy, developed ileitis during follow up, and most probably was wrongly diagnosed as UC at diagnosis (37, 38).

Despite presentation with severe disease at a very young age, none of our patients died. Mortality has rarely been reported in relation to IO-IBD. However, higher mortality rates, 7% and 19% were reported for IO-IBD cohorts with high proportions (17% and 44%) of monogenic diseases and immune deficiency (5, 39). The small size of our series precluded identifying factors that associate with such favorable outcomes as long-term remission and treatment de-escalation.

The variable outcomes between studies on IO-IBD and VEO-IBD may result from differences between the cohorts in the study periods, the availability of biologic treatment, the extent of genetic evaluations, and the proportions of patients with monogenic diseases.

Our study has limitations that are associated with its multicenter retrospective design. The genetic evaluation and treatment of VEO-IBD in general, and particularly of IO-IBD, have changed significantly over the past two decades. Secondly, our definition of remission was clinical, and did not include fecal calprotectin levels or consistent evaluation of endoscopic remission. In addition, small bowel imaging was not feasible in this age group at presentation. Nevertheless, our study contributes information on the long-term outcomes of children with IO-IBD as a distinct group, rather than as a part of VEO-IBD and shows similar results to data from a validated Israeli IBD Research Nucleus (29), thus providing validity to our results.

In conclusion, though the evaluation and treatment of children with IO-IBD pose substantial challenges, a large proportion of children have favorable long-term prognosis, even following severe disease course at presentation. Prospective studies of this population are needed to identify predictors for favorable outcomes, to identify those with monogenic diseases, and to evaluate responses to biological treatment using therapeutic drug monitoring.

## Data Availability

The original contributions presented in the study are included in the article, further inquiries can be directed to the corresponding author.
